# Tension Capacity of Crushed Limestone–Cement Grout

**DOI:** 10.3390/ma17153860

**Published:** 2024-08-04

**Authors:** Muawia Dafalla, Ahmed M. Al-Mahbashi, Ahmed Alnuaim

**Affiliations:** Civil Engineering Department, College of Engineering, King Saud University, P.O. Box 800, Riyadh 11421, Saudi Arabia; aalmahbashi@ksu.edu.sa (A.M.A.-M.); alnuaim@ksu.edu.sa (A.A.)

**Keywords:** limestone, grout, stress–strain relationship, compressive strength

## Abstract

The feasibility of using crushed limestone instead of sand in cement grout is examined in this work. This study entails performing several tests, including the Brazilian test, the compressive strength test, and the stress–strain correlation test. The curing times used were 7, 14, and 28 days for mixtures with various proportions of cement to limestone (1:1, 1:2, and 1:4). The conventional sand–cement grout laboratory tests were prepared using a similar methodology to examine the effectiveness of the suggested substitute. The findings show that the limestone-based grout has sufficient strength, but that it is less than that of the typical sand material. The values of the tensile strength and elastic modulus were determined. A focus was made on the tensile strength and stress–strain relationship. A special laboratory set-up was used to look at the progress of failure using strain gauges fitted to the cylindrical samples both vertically and horizontally. The angular shape of the particles’ ability to interlock is responsible for the material’s increase in strength. According to this study, crushed limestone can be used as a substitute for sand in circumstances where sand supply is constrained. The suggested grout can be used in the shotcrete of tunnels and rock surfaces.

## 1. Introduction

The Brazilian test, which is also known as the indirect tensile test, is often used to determine the ability of materials to resist tension. The evaluation results of the stress–strain relationship and the failure properties obtained by this test are of great significance in the assessment of construction materials. This study is aimed at investigating the use of crushed limestone in cement grout for the stabilization and shotcrete treatment of tunnels. Previous research has shown that grouting techniques based on cement could effectively protect concrete structures from deterioration due to corrosion in steel bars [1] or the development of cracks in tension zones [2]. In addition, this material could be used to mitigate or repair the developed cracks in infrastructure construction exposed to static or dynamic loads [3]. In special applications, cement–water grout may be required for several reasons such as enhancement of the basic properties such as strength and tension resistance. The introduction of the metro transportation system to Riyadh City during the last five years has resulted in large amounts of excavated materials [4]. Tunnel boring machine technology was employed to construct the proposed tracks. The spoil material is dominantly crushed limestone. The majority of these components are variously graded crushed limestone. These materials have been employed in several geotechnical applications, including fluid management in clay liners [5], green structural concrete aggregation [4], and a reduction in the usage of commercial bentonite to be ecologically friendly [6].

Cement-based grout is primarily used in tunnel lining, crack repair, soil enhancement (jet grouting), and concrete applications. It is created by combining cement, sand, and water, with the addition of admixtures on some occasions [7,8].

Because limestone aggregates are efficient and reasonably priced, their usage in the concrete industry has been growing [9]. Aquino et al. [10] investigated the effects on the characteristics of concrete of varying ratios of fine limestone to sand at varying water-to-cement ratios. The obtained results demonstrate that adding more finely ground limestone to the concrete mixture increases the concrete’s flexural strength, compressive strength, and elasticity modulus. Burhan Alshahwany [11] assessed how some characteristics of regular concrete were affected when sand was substituted with limestone filler. Additives to cement–water grout have been used to improve workability or to achieve the required properties of strength and resistance to tension cracks [12,13,14]. Shannag [13] reported that the use of natural materials such as pozzolan with silica fume has a significant role in producing a high-performance cement-based grout for special use in the concrete industry.

The findings indicate that adding up to 20% more limestone to the sand does not influence the strength of the concrete. Conversely, the scarcity of natural sand in many regions of the world has the potential to impede the expansion of the concrete industry’s demand. For this reason, crushed limestone has been proven to be a successful substitute for natural sand. Compared to concrete–sand-based combinations, the concrete made with crushed limestone requires more water for mixing [15]. The mix design of the grout for the tunnel lining requires an understanding of the environment and stresses within the close boundaries of the tunnel opening. Tension stresses may be created within the rock mass or the broken parts along the rim or edges of the excavation. The gradation, particle size distribution, density, compaction, and flowability are very important factors in establishing a grout mix design. Knowledge of these parameters will help in supplying durable materials. The mix design of the grout should always consider its ability to resist tension to achieve stable, sound, and durable grout.

The limestone filler was proved to increase the degree of hydration. Bonaveet et al. [16] stated that the use of limestone filler is a rational option for reduced energy consumption and emission reduction. They found that the optimum level of limestone addition depends on the concrete mixture proportions. Wang et al. [17] conducted a review on the effects of the addition of limestone powder on the properties of concrete and confirmed that the use of limestone powder to replace fine aggregate improves the properties of concrete. Grout, known as highly fluid concrete that flows under its own weight or pressure, can be simulated to self-consolidated concrete (SCC). Daoud and Mahgoub [18] studied the addition of limestone powder to self-consolidated concrete and found that the addition of 30% of the cement weight can reduce the cost and enhance the performance of SCC in terms of workability and flowability. A similar outcome is presented by Valcuende et al. [19] who confirmed the speeding up of the hydration reaction and that the higher the fine limestone content in the mix, the shorter the initial and final concrete setting times. Properties including compression strength, splitting tensile, and workability were apparently improved due to the addition of crushed limestone dust to concrete [20].

The goal of this study is to look into how a finely ground, crushed limestone powder that is taken out of a TBM could be used as a cement grout material for shotcrete and tunnel lining repair. The present study also provides an assessment of the engineering properties and tension capacity of the grout. The recommended mixture may serve as a cost-effective substitute for lean concrete or sand–cement grouts, which are employed in tunnel lining. With a reduced need for cement and crushed sand, this material should be both economical and environmentally benign.

The Brazilian test and tension capacity are examined in this study for suggested mixtures where sand is entirely replaced with crushed limestone material. The elasticity and compression behavior are presented along with the obtained failure mode.

## 2. Materials and Methods

### 2.1. Materials

The primary ingredients in the mixtures employed in this investigation include cement (c), sand (s), and crushed limestone. The limestone (LS) was obtained from Riyadh, Saudi Arabia, and is intended as a substitute or replacement for sand in grout mixtures. This limestone is a spoil crushed material produced by tunnel boring machines. The percentages of the limestone material used in the mixtures compared to cement are shown in Table 1. In this investigation, three percentages of limestone aggregates, 1, 1.2, and 1.4 of the dry weight of cement content, were taken into consideration. The effectiveness of employing the crushed limestone material in place of sand was compared and evaluated using four different mixtures, the fourth of which is a control mixture of cement and sand (1:1). For blending and combining cement with limestone or sand, distilled water was used.

**Figure 1:**
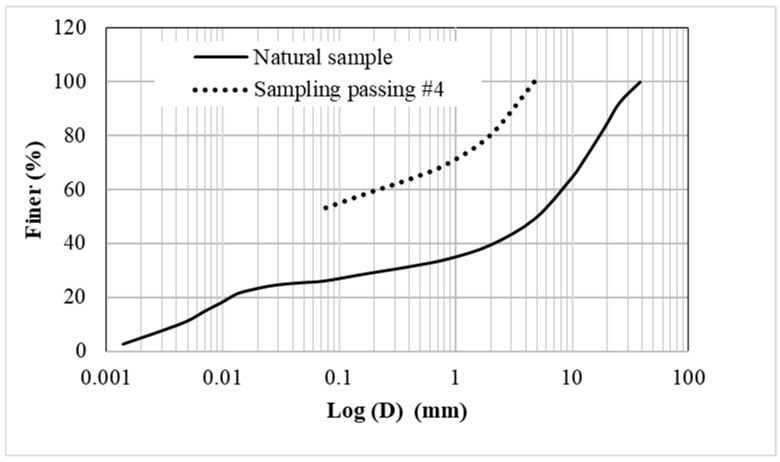
The particle size distribution of the crushed limestone.

In this study, Yamama Portland cement, which was produced locally in KSA (Alkharj Plant Sation), was used. The sand used in this study is common construction local sand and is classified as poorly graded (SP) in accordance with ASTM D2487-17 [21,22]. The sand origin is the Al-Thumamah area east of Riyadh, which is overlain by a huge reserve of windblown and subsurface sand deposits. The coefficient of uniformity and the coefficient of concavity are 1.713 and 0.945, respectively. The sand was substituted with different amounts of crushed limestone (obtained from the tunnel boring equipment) that passed through sieve number 4. Figure 1 presents the grain size distribution for the crushed limestone, including the sample passing through #4, used in the mixtures.

The natural sample refers to the sample obtained from the site. Screening using a 3/4” sieve gave a material referred to as natural soil passing through sieve 3/4”. The material passing through sieve #4 is the material used in the grout mixture.

### 2.2. Mixture Preparation

The materials of cement, sand, and crushed limestone were prepared based on the mixture percentages shown in Table 1. The sand and limestone materials were allowed to dry in an oven for a day. All mixtures were mixed in a dry condition, and then, a predetermined amount of distilled water was added to the mixture, which was then thoroughly mixed in a suitable pan. First of all, the control mixture with a cement–sand composition was prepared. Three different percentages of the limestone material as a proportion of the cement were added to compose the targeted mixtures. The amount of distilled water added to the mixtures was estimated to be within the ratio of 0.6 for the water-to-cement content. One exception is that for the cement–sand mixture, this ratio was reduced by 10 percent for workability considerations, and the final results were adjusted using the appropriate curves to be the equivalent of the same water/cement ratio.

### 2.3. Specimen Preparation

**Figure 2:**
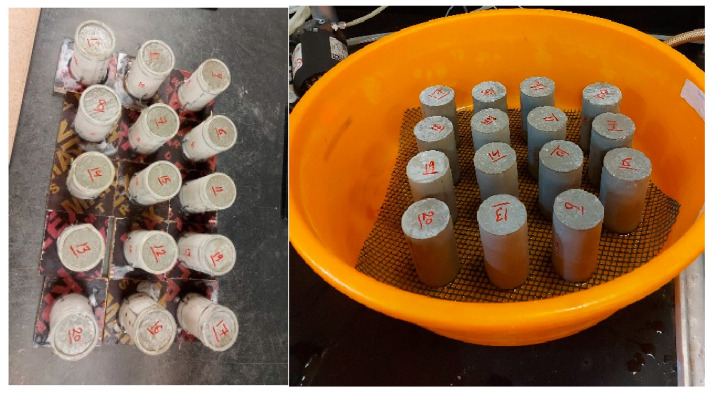
The prepared specimens after casting.

The prepared mixture in the previous section was cast into appropriate molds. As shown in Figure 2, molds measuring 100 mm in height and 50 mm in diameter were built. A longitudinal groove was created to make it easier to extrude the specimen, and it was filled with silicon grease to stop water leakage after casting. A thin layer of oil was applied to coat the interior wall of the mold; this step was necessary to eliminate friction during extruding the specimen. Three to five layers of mixture were cast onto the mold, and each layer was compacted with a steel rod to ensure a homogeneous specimen without air voids. A smooth-edged knife was used to finish each specimen’s upper surface.

The specimens were extruded from the casting molds after being kept at room temperature throughout the night. A special water basin was prepared, and the specimens were transferred and placed above plastic mesh to ensure water accessibility (Figure 2). The basin was filled with distilled water and the specimens were kept for curing periods of 7, 14, or 28 days.

### 2.4. Testing Procedure

**Figure 3:**
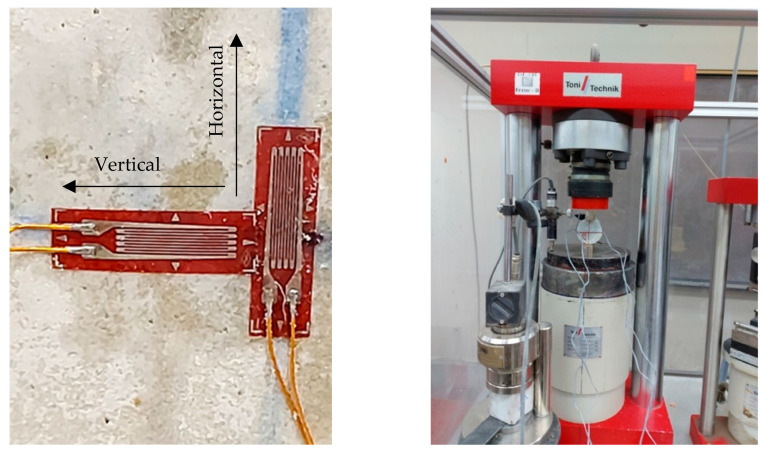
Fitted strain gauges and compression machine set-up.

By the end of the scheduled curing periods of 7, 14, or 28 days, the specimens were taken out of the water basin, and the excess water was removed from the specimens. The dimensions for each specimen, including height and diameter, were measured at different levels (i.e., top, middle, and bottom) and the average values for two heights and three diameters were considered, with the weight of each specimen also recorded. The smoothness of the tested specimens was checked before performing the Brazilian test. Rough sandpapers were used to ensure that irregularities across the surfaces were less than 0.25 mm. Figure 3 shows the 3 MN Toni/Technik compression machine that was employed in this investigation. An inbuilt transducer allows the machine to measure the displacement and control the rate of vertical deformation. An external data logger equipped with additional strain gauges (5 mm) was used to register the strain in two directions along the exterior surface of the cylindrical specimen during the test. The stress–strain curve for each specimen can be drawn using this system. 

The specimens were then placed in the compression machine, as shown in Figure 3.

A low rate was applied at 0.09 kN/s. The nominal range of the loading rate according to each specimen’s size and specifications ranged between 0.09 kN/s and 0.18 kN/s. Alokili et al. [23] present in more detail the testing procedures followed in this study and a brief description of the compression and crushing strength of the limestone–cement grout.

## 3. Results and Discussion

### 3.1. Compressive Strength over Examined Curing Periods

Table 2 presents the test results of the compressive strength for the specimens tested at specified curing times.

### 3.2. Plots of Load vs. Deformation

**Figure 4:**
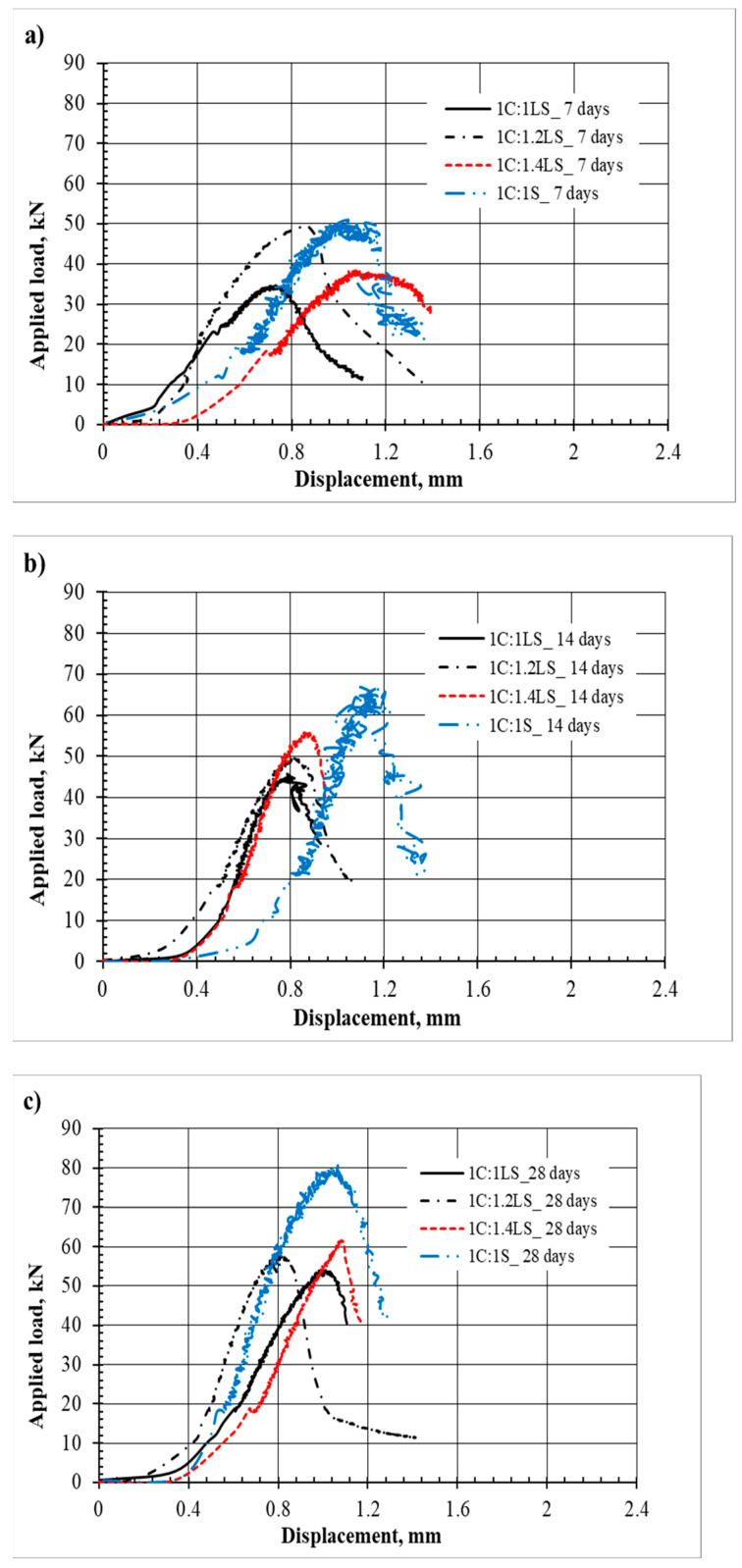
Load versus deformation at curing times of (**a**) 7 days, (**b**) 14 days, and (**c**) 28 days.

The vertical load versus deformation curves for the compression tests are presented here to help us understand the progress and development of cracks and the failure of the newly introduced limestone–cement grout mixture and the standard sand–cement grout (Figure 4).

The load–displacement data and charts presented here are aimed at showing the general trends of the load–displacement relationships and may not be taken as a quantitative measure.

### 3.3. Modulus of Elasticity (E)

The calculation of the modulus of elasticity was performed in accordance with ASTM D3148-96 [24]; it represents the ratio between the variation in stress (∆σ) and the variation in strain (∆ε) from the stress–strain curve. The slope of the straight line portion of each specimen’s stress–strain curve is known as the average Young’s modulus or E_avg_. The Secant Young’s modulus (E_secant_) is the slope of the stress–strain line from the origin to a specific percentage of the ultimate strength, typically estimated to be between 50 and 75% of the ultimate strength. Table 3 presents the calculated moduli of elasticity (i.e., E_secant_ and E_avg_).

### 3.4. Modes of Failure

**Figure 5:**
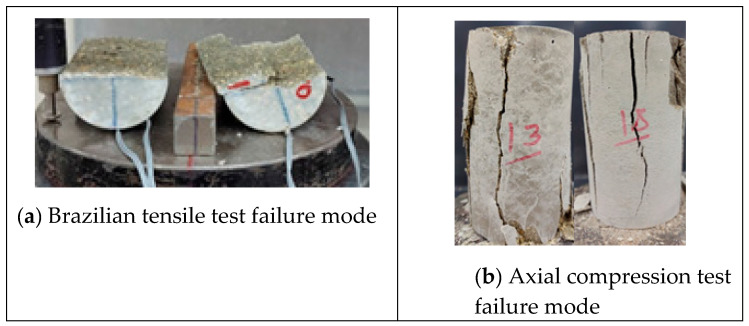
Typical failure mode for tensile and compression tests of limestone–cement grout.

**Figure 6:**
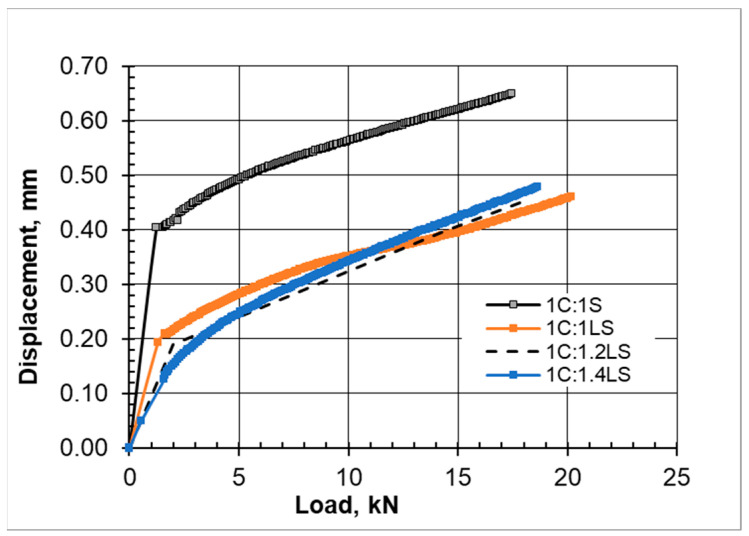
Vertical displacement versus load in Brazilian tests.

**Figure 7:**
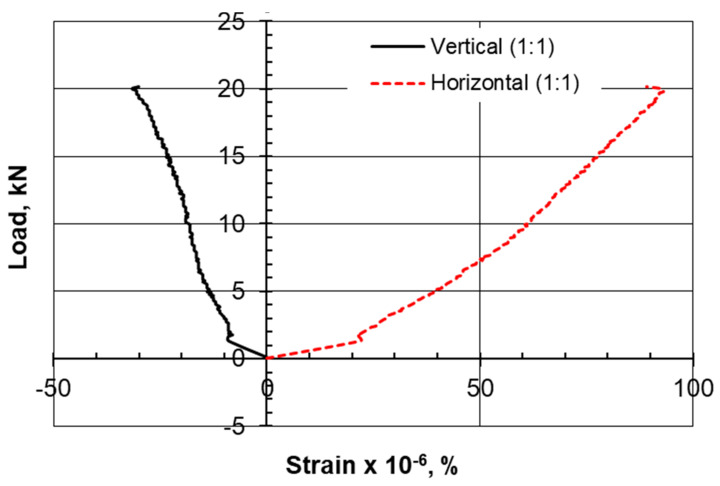
Vertical and lateral strains during split tests (strain gauges), 1C:1LS.

**Figure 8:**
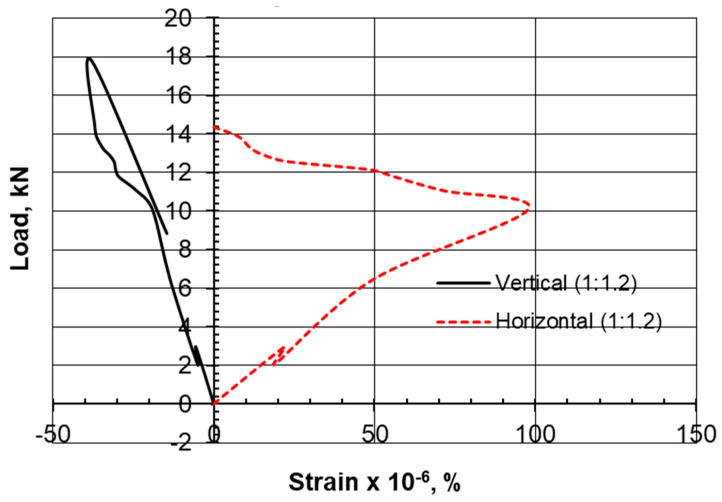
Vertical and lateral strains during split tests (strain gauges), 1C:1.2LS.

**Figure 9:**
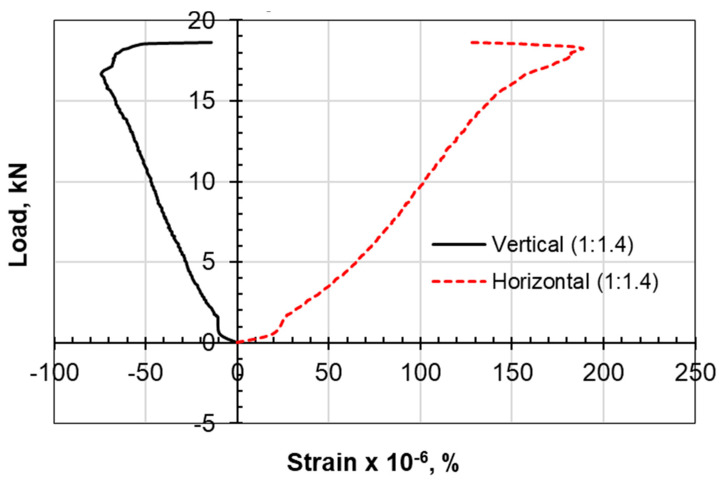
Vertical and lateral strains during split tests (strain gauges), 1C:1.4LS.

**Figure 10:**
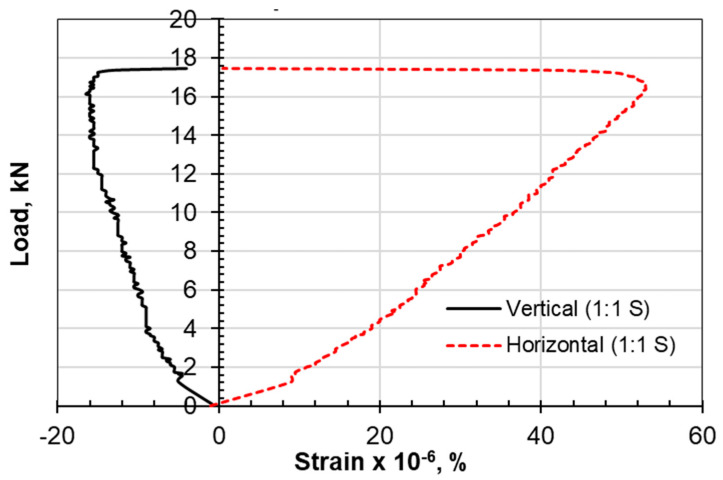
Vertical and lateral strains during split tests (strain gauges), 1:1 sand/cement.

The type of failure and deformation that took place during the testing of the specimens for the compression tests as well as the Brazilian tensile test for the limestone–cement grout is shown in Figure 5. The rupture took place along defined, clear vertical planes. Multiple shear failure planes are observed for the compression test, and a single central plane is noted for the Brazilian tensile test. Negative lateral stresses acting perpendicular to the loading vertical axis are responsible for splitting the cylindrical specimen into two equal parts, with a plane of failure that is not uniform or smooth due to the presence of the non-uniform crushed aggregate of limestone. Vertical displacement versus load in the Brazilian tests is shown in Figure 6. Vertical and lateral strains during split tests (strain gauges) are shown in Figure 7, Figure 8, Figure 9 and Figure 10.

The compressive strength obtained at three different curing times indicates that 60 to 84% strength is gained in the first 7 days, which is not different from the levels obtained for concrete. The compressive strength of limestone–cement grout is in the order of 72% compared to the sand–cement grout. This is due to the reduced water/cement ratio used in the sand–cement grout mix. This can indicate that the use of crushed limestone instead of sand may not reduce the compressive strength.

## 4. Discussion

The Brazilian test conducted in accordance with ASTM C496/C496M-17 [25] or ASTM D3967-16 [26] is generally computed using the formula in Equation (1):

σ_t_ = 2* P/(π* D*t). (1)

D is the diameter of the specimen, t is the width of the specimen, and P is the recorded load.

This is reduced to

σ_t_ = 0.636 ∗ P/D*, (2)

where D is the diameter of the tested sample and t is the width of the sample (Equation (2)). The computed tensile strength obtained at failure is given in Table 4.

It can be observed that the tensile strength does not improve with a greater addition of crushed limestone, unlike the compressive strength, where an increase from 25.97 kN/m^2^ to 30.05 kN/m^2^ was observed when the cement/limestone ratio was increased from 1 to 1.4.

The majority of the elasticity modulus variation is negligible and centers on the values of the specimens that belong to the cement–sand combination.

An increase in the limestone material results in an increase in strength when analyzing the evolution of compressive strength over the curing period. The growing compressive strength of 90% of the cement–sand control mixture is indicated by the percentage of the limestone material (1.1). The angular shape of the particles interlocks, which is responsible for the strength increase in the proposed limestone material mixes.

This shows that in situations where sand is scarce, using this limestone material can effectively replace the sand. For combinations with a greater percentage of limestone (1.4) and for the control mixture, this range has exceeded 1.5%. The strain at the ultimate load and failure is generally found between 0.5% and 1%.

**Figure 11:**
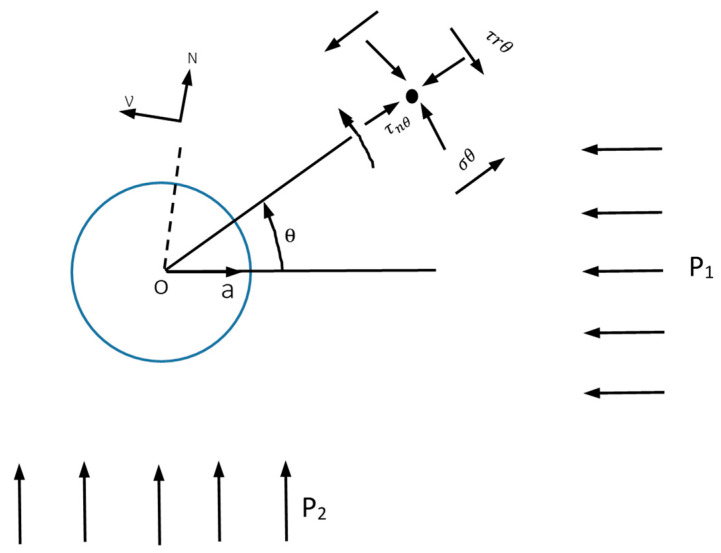
The radial and tangential stresses for a circular hole in an infinite disk [27].

The procedure for split testing is enhanced with strain gauges at the exterior surface of the cylindrical samples. An analytical approach using the Kirish solution (Figure 11) for a biaxially loaded plate of homogeneous, isotropic, and elastic material with a circular opening is the most widely used method for determining the induced stresses [27].

The concept of plain strain can estimate the radial and tangential stresses using Equations (3)–(5):

σ_r_ = 0.5 (P_1_ + P_2_) (1 − a^2^/r^2^) + 0.5 (P_1_ − P_2_)(1 − 4a^2^/r^2^ + 3a^4^/r^4^) cos2θ, (3)

σ_θ_ = 0.5 (P_1_ + P_2_) (1 + a^2^/r^2^) − 0.5 (P_1_ − P_2_) (1 + 3a^4^/r^4^) cos2θ, (4)

τ_r θ_ = −0.5 (P_1_ − P_2_) (1 + 4a^2^/r^2^ − 3a^4^/r^4^) sin2θ. (5)

Considering that the inner opening circle is an extremely small part of the stresses where the strain gauges are fitted, θ = 0, P_1_ = 0, and a = 0.

Equations (3)–(5) lead to Equations (6)–(8):

σ_r_ = 0.5 (P_2_) + 0.5 (−P_2_) (6)

σ_θ_ = 0.5 (P_2_) − 0.5 (−P_2_) (7)

τ_r θ_ = 0 (8)

when a is equal to r, and σ_θ_ can increase to 3 times the axial pressure (3P_2_). This solution applies to tunnels with a circular opening rather than a solid core subjected to stresses.

The axial stresses are always associated with bulging, and a negative lateral stress causes the cylindrical specimen to increase in the lateral dimension until failure occurs. The computational approaches using finite elements are very useful, despite the fact that the assumed shape keeps changing to an ellipsoidal geometry of varying definition. The exact profile of changes can be obtained experimentally for solid cylindrical specimen materials, excluding all approximations made in mathematical solutions.

Previous studies on the crack initiation of rock material conducted by Nicksiar and Martin [28], Tao et al. [29], and Mutaz [30] defined four zones of rock core cracking under stress. The first zone is defined as crack closure, followed by a crack initiation zone where distress is caused by shear forces and distortion in planes. The third zone is an area of instability and the growth of cracks, and the fourth zone is the peak resistance and failure zone. The crack initiation zone was claimed to start at 30% to 60% of the uniaxial compressive strength. In this study, it was decided to compare two perpendicular strains at the outer side of the cylindrical specimen diameter and observe the sample integrity at each stage.

Examining the horizontal-to-vertical strain ratio at the outer surface of the core, it can be observed that the growth of the ε_h_/ε_v_ ratio from the stable elastic zone by 10% can move the status of the stress–strain relationship to the crack initiation zone or failure. The stable elastic zone is assumed when the bilinear stress–strain response ceases. The choice of 25% failure load is believed to be within the stable elastic zone.

Lajtai [31] suggested that lateral strain is the prime indicator for crack initiation. According to this reference, the lateral strain on the stress–strain response is more sensitive to the development of cracks than the axial strain is, even before unstable crack propagation begins. As a result, the point at which the lateral strain diverges from the linearity of the axial stress ($\sigma_{1}$) vs. the lateral strain curve is identified as crack initiation ($\sigma_{ci}$).

The initiation of cracks, according to the lateral strain model, takes place at a higher level for the limestone–cement grout compared to the sand–cement grout. The 1:1.4 ratio of the limestone–cement grout scored a stable lateral strain of 150, which is three times the crack initiation level (CI) of that established for the 1:1 sand–cement grout (50).

However, the authors suggest considering the strain in the perpendicular direction as a factor. It was observed that when the ε_h_/ε_v_ ratio is increased by 10%, tension failure is reached (Table 5, Table 6, Table 7 and Table 8).

The axial strain at failure in the sand–cement grout is found to be 1.2 for the compression test, while this strain is reported to be from 0.8 to 1.0 for the limestone–cement grout replacement. In the Brazilian tensile test, it was also observed that the axial strain along the loading axis is much higher than the strain for the limestone–cement grout material.

**Figure 12:**
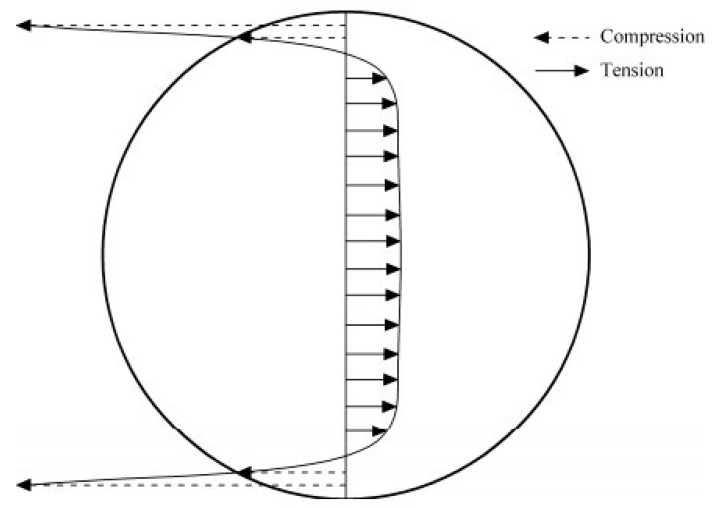
Stress distribution along the loading axis of a Brazilian core test [25].

The tensile stress is highest along the loading axis, and failure occurs by splitting along the same axis, unlike compression test failures where multiple vertical shear plane failures occur (Figure 12).

The lateral strain model combined with the ε_h_/ε_v_ growth are good predictors for crack initiation in shotcrete or grout material. The sand can be successfully replaced by crushed limestone materials for shotcrete grout material in tunnels and underground utilities or other applications.

When comparing the compressive strength of the limestone–cement grout to that of the sand–cement grout, the low moisture/cement ratio used in the conventional sand–cement material must be corrected for.

A water/cement ratio of 0.48 can achieve a compressive strength of 27.6 MPa, while a water/cement ratio of 0.59 can provide a compressive strength of 20.7 MPa, as quoted by Sidney M. Levy [32]. This implies an increase in the measured strength for the sand–cement grout by about 25% when compared with other grout groups mixed at a 0.60 moisture/cement ratio. The percentage of the limestone material of 1.1 showed a compressive strength in the range of 90% of the control mixture of cement–sand. Comparing other mixtures in which the aggregate/cement ratio is different from 1:1 needs to be corrected for if reliable numbers are required.

Abdelgader and Elgalhud [33] presented many formulas to express relationships between the compressive and tensile strengths of grout materials, but these were not found to be representative of grouts made of crushed limestone. The experiments conducted in this study yielded values of split tension capacities ranging from 2.27 to 2.54 N/mm^2^. This can be attributed to the type of cement, testing conditions, and other factors. The figures obtained in this study are in agreement with the typical split tension capacity of grout and low-strength concrete.

## 5. Conclusions

This study is focused on the initiation of cracks and failure in a limestone–cement grout material proposed as an alternative to sand–cement grout. It was found that crushed limestone can be utilized successfully as a shotcrete material with satisfactory performance with regard to compressive strength and tensile strength capacity. The percentage of the limestone material of 1.1 showed a compressive strength in the range of 90% of the control mixture of cement–sand. The 1:1.2 and 1:1.4 mixtures demonstrated high compressive strength values exceeding that of the control mixture. The crack initiation model based on lateral strain can be examined using the ratio of ε_h_/ε_v_ to assess crack initiation and failure zones. It was observed that when the ε_v_/ε_h_ ratio is increased by 10%, tension failure is reached. In the Brazilian tensile test, it was also observed that the axial strain along the loading axis is much higher than the strain for the limestone–cement grout material. The 1:1.4 ratio of limestone–cement grout scored a stable lateral strain of 150, which is three times the crack initiation level (CI) that is established for the 1:1 sand–cement grout (50). The split tensile strength of the limestone–cement grout was found to be equal to or higher than that of the conventional sand–cement grout.

It can be concluded that the crushed limestone material can replace sand in grout mixtures and perform well within the range of compressive and tensile stresses when reported within the design range specified. Crack initiation occurs at higher stresses when the limestone/cement ratio is 1.4. This study is limited to the common limestone rock material found in the Riyadh area and other sedimentary rocks. Rocks of different origin need to be investigated using a similar approach. Impacts on long-term performance and environmental effects are suggested for future studies. Considering the use of a plasticizer in future studies can be useful to manage the water/cement ratio of the grout mixture.

## Figures and Tables

**Table 1:** The cement, limestone, and sand mixtures adopted in this study.

Mixture No.		1	2	3	4
Material ratio	Cement, C	1	1	1	1
	Limestone, LS	1	1.2	1.4	-
	Sand, S	-	-	-	1
	Water/cement ratio	0.6	0.6	0.6	0.6 *
Specimens	Curing period (days)	7	7	7	7
		14	14	14	14
		28	28	28	28

* Adjusted for workability issues.

**Table 2:** Compressive strength in MPa for three curing periods.

Specimens		1	2	3	4
Material portions	Cement, C	1	1	1	1
	Limestone, LS	1	1.2	1.4	-
	Sand, S	-	-	-	1
Curing in days	7	16.620	23.710	18.320	24.940
	14	21.980	24.090	26.870	32.440
	28	25.970	28.130	30.050	39.140

**Table 3:** Secant modulus of elasticity (E_secant_ and E_avg_) for all mixtures at variable curing times.

Mixture	Secant Modulus of Elasticity (Esecant) GPa			Average Modulus of Elasticity (Eavg) GPa		
	Curing Days			Curing Days		
	7	14	28	7	14	28
1C:1LS	22.59	31.34	27.36	32.50	27.27	29.45
1C:1.2LS	47.72	23.73	33.70	30.56	33.09	21.09
1C:1.4LS	22.22	43.75	33.59	22.43	32.50	27.73
1C:1S	20.31	32.44	57.20	17.43	44.44	57.14

**Table 4:** Split test results.

Mixture	(1C:1 LS)	(1C:1.2 LS)	(1C:1.4 LS)	(1C:1 Sand)
Max load kN	20.15	17.81	18.63	17.46
S_pt_ N/mm^2^	2.54	2.27	2.38	2.29

**Table 5:** Horizontal/vertical strain ratio at four stages of loading, 1C: 1LS.

Stage of Loading Relative to Failure Load (20.15 kN)	Vertical Strain ε_v_ (10^−6^)	Horizontal Strain ε_h_ (10^−6^)	Horizontal/Vertical Strain Ratio ε_h_/ε_v_
25%	−15	40	2.67
50%	−20	62	3.1
75%	−25	80	3.2
100%	−30	90	3.75

**Table 6:** Horizontal/vertical strain ratio at four stages of loading, 1C:2LS.

Stage of Loading Relative to Failure Load (17.81 kN)	Vertical Strain ε_v_ (10^−6^)	Horizontal Strain ε_h_ (10^−6^)	Horizontal/Vertical Strain Ratio ε_h_/ε_v_
25%	−10	38	3.8
50%	−20	85	4.25
75%	−35	-	Failure range
100%	−37	-	Failure range

**Table 7:** Horizontal/vertical strain ratio at four stages of loading, 1C:1.4 LS.

Stage of Loading Relative to Failure Load (18.63 kN)	Vertical Strain ε_v_ (10^−6^)	Horizontal Strain ε_h_ (10^−6^)	Horizontal/Vertical Strain Ratio ε_h_/ε_v_
25%	−25	58	2.32
50%	−43	100	2.32
75%	−65	140	2.5
100%	-	170	Failure zone

**Table 8:** Horizontal/vertical strain ratio at four stages of loading, 1C:1S.

Stage of Loading Relative to Failure Load (17.46 kN)	Vertical Strain ε_v_ (10^−6^)	Horizontal Strain ε_h_ (10^−6^)	Horizontal/Vertical Strain Ratio ε_h_/ε_v_
25%	−10	24	2.40
50%	−14	34	2.42
75%	−18	46	2.55
100%	−17	54	3.20

## Data Availability

The data are contained within the article.
